# From covalent bonding to coalescence of metallic nanorods

**DOI:** 10.1186/1556-276X-6-559

**Published:** 2011-10-25

**Authors:** Soohwan Lee, Hanchen Huang

**Affiliations:** 1 Department of Mechanical Engineering, University of Connecticut, Storrs, CT 06269, USA

**Keywords:** metal surface steps, adatom, diffusion, covalent bonding, simulation

## Abstract

Growth of metallic nanorods by physical vapor deposition is a common practice, and the origin of their dimensions is a characteristic length scale that depends on the three-dimensional Ehrlich-Schwoebel (3D ES) barrier. For most metals, the 3D ES barrier is large so the characteristic length scale is on the order of 200 nm. Using density functional theory-based *ab initio *calculations, this paper reports that the 3D ES barrier of Al is small, making it infeasible to grow Al nanorods. By analyzing electron density distributions, this paper shows that the small barrier is the result of covalent bonding in Al. Beyond the infeasibility of growing Al nanorods by physical vapor deposition, the results of this paper suggest a new mechanism of controlling the 3D ES barrier and thereby nanorod growth. The modification of local degree of covalent bonding, for example, via the introduction of surfactants, can increase the 3D ES barrier and promote nanorod growth, or decrease the 3D ES barrier and promote thin film growth.

## Introduction

The growth of metallic nanorods by physical vapor deposition (PVD) is a common practice. Limited diffusion is a critical condition for nanorod growth [1], and geometrical shadowing in glancing angle deposition further promotes nanorod growth [2,3]. In addition to the diffusion of adatoms on flat surfaces, when diffusing over monolayer surface steps, adatoms experience a large energy barrier--he Ehrlich-Schwoebel (ES) barrier--which affects the resulting surface [4,5]. Adatoms diffusing over multiple-layer steps experience even larger energy barriers [6-9]; this barrier is referred to as three-dimensional (3D) ES barrier; for comparison, the conventional ES barrier is called the two-dimensional (2D) ES barrier. Further, even small clusters experience 3D ES barriers [10], and variations of 3D ES barriers exist when steps intersect [11,12].

The 3D ES barriers for metals are much larger than their 2D counterparts, for example, 0.40 versus 0.16 eV for Cu [8]. These 3D ES barriers, when sufficiently large, stabilize multiple-layer surface steps, which in turn enable the effective operation of the 3D ES barriers [13]. The dynamic competition of multiple-layer and monolayer surface steps and consequently the competition of diffusion over 3D and 2D ES barriers give rise to the characteristic length scale of nanorod dimension [14]. When this length scale is too large, larger than 1 μm, the growth of nanorods becomes unfeasible.

It is worth noting that the characteristic length scale of nanorods becomes much larger than 1 μm, when the 3D ES barrier is reduced from 0.40 to 0.16 eV [14]. A smaller 3D ES barrier is possible when covalent bonding, even in metals such as Al [15], becomes important. For metallic surfaces, the large 3D ES barrier is the result of low coordination of a diffusing adatom at the saddle point. In comparison, covalent bonding can lower the energy state of atoms even with low coordination. As an example, a bulk silicon atom has only four nearest neighbors, in contrast to 12 nearest neighbors of face-centered cubic (FCC) metals. Even in metals, some degree of covalent bonding exists. This is the case for Al, which has *s *and *p *outer electrons. The covalent nature of Al bonding has been found to be responsible for its ultra large ideal shear strength [15]. Even though the shear modulus of Al is lower than that of Cu, the ideal shear strength of Al is higher than that of Cu. The covalent nature of Al bonding is also responsible for its anomalous outward relaxation of {111} and {100} surfaces [16]. In contrast, similar surfaces of most metals exhibit inward relaxation due to the missing coordination and electrons at surfaces [17,18].

The logic connection from covalent bonding to small 3D ES barrier and then to increased characteristic length scale of growing nanorods may apply to Al. In contrast to numerous reports of Cu nanorods using PVD [2,3,19], there is the lack of report on Al nanorods using the same method. In appearance, Cu and Al have comparable melting temperatures and sublimation energies. One might expect similar feasibility of Cu and Al nanorods using PVD.

Based on the established logic connection, we hypothesize that the PVD growth of Al nanorods is not feasible without substrate cooling due to the small 3D ES barrier of Al. To verify our hypothesis, we use *ab initio *calculations to show that the 3D ES barrier of Al is substantially smaller than the value of Cu and that the smaller barrier correlates with the covalent bonding of Al. Taking this result one step further, we suggest that one can perturb the local degree of covalent bonding by introducing an impurity atom. If such atom also tends to float on the surface, this use of impurity atoms represents a new mechanism of surfactant application, the new mechanism being the control of local covalent bonding.

## Simulation method

We describe the research method of this study--density functional theory-based *ab initio *calculations. Our calculations rely on the ABINIT package, which uses a plane-wave basis [20,21] within the local density approximation of the norm-conserving Troullier-Martins pseudopotentials [22]. We choose the cutoff energy in plane-wave expansion as 300 eV, for convergence within 0.01 eV [23]. The valence electrons of Al in ABINIT are 3*s*^2^3*p*^1^. The *k*-point meshing follows the Monkhorst-Pack scheme with 2 × 4 × 1 grids [24].

**Figure 1:**
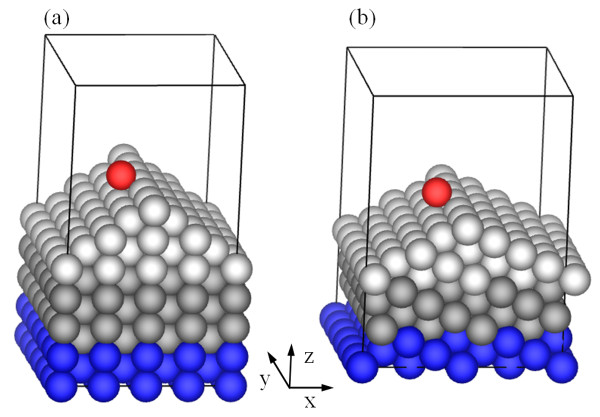
**A simulation cell**. It contains (a) a B-type step and (b) an A-type step, with vacuum region above the adatom.

In setting up the supercell, we focus on the configuration of multiple-layer (four-layer) steps, but we also examine configurations of steps with fewer layers in thickness. Figure 1a is the supercell for a B-type <110> step of four layers, and it contains 277 atoms. The adatom diffuses from the left {111} facet to the right {111} facet over a four-layer step. One horizontal *x *direction is [100] and the other horizontal *y *direction along the step is [$0\bar{1}1$], making the vertical *z *direction to be [$0\bar{1}\bar{1}$]. There are four double layers along *x *(1.5932 nm), six double layers along *y *(1.6898 nm), and six double layers in the flat substrate along *z *(1.6898 nm); the vacuum region is equivalent to five double layers, above the adatom; it is 1.4082 nm. To mimic a bulk environment, atoms (blue in Figure 1) in the three single layers at the bottom are fixed to their bulk positions. When the vertical (*z*) dimension is increased by four single layers, the results converge within 0.01 eV; when the dimension of the *x *direction is increased by four single layers, the results converge within 0.02 eV; and when the *y *direction is changed by two single layers, the results do not change. To calculate the diffusion barriers, we use the nudged elastic band method with eight images as in our previous studies [8].

Based on the convergence tests, we use comparable dimensions in all calculations of various steps. When the B-type step is three layers, the *x*, *y*, and *z *directions are [$\bar{1}\bar{1}\bar{8}$], [$0\bar{1}1$], and [$\bar{7}\bar{1}\bar{1}$], respectively, so periodic boundary conditions along the horizontal directions still apply, and the cell contains 277 atoms. When the B-type step is two layers, the *x*, *y*, and *z *directions are [$\bar{1}\bar{1}4$], [$0\bar{1}1$], and [$\bar{3}\bar{1}\bar{1}$], respectively, and the cell contains 277 atoms. When the B-type step is a monolayer, the *x*, *y*, and *z *directions are [$\bar{3}\bar{3}8$], [$0\bar{1}1$], and [$\bar{5}\bar{3}\bar{3}$], respectively, and the cell contains 277 atoms. Figure 1b is the supercell for an A-type <110> step of four layers with the *x*, *y*, and *z *directions being [332], [$0\bar{1}1$], and [$\bar{5}33$], respectively, and it contains 253 atoms. When the A-type step is three layers, the *x*, *y*, and *z *directions are [111], [$0\bar{1}1$], and [$\bar{2}11$], respectively, and the cell contains 289 atoms. When the A-type step is two layers, the *x*, *y*, and *z *directions are [556], [$0\bar{1}1$], and [$\bar{11}55$], respectively, and the cell contains 253 atoms. When the A-type step is a monolayer, the *x*, *y*, and *z *directions are [558], [$0\bar{1}1$], and [$\bar{13}55$], respectively, and the cell contains 289 atoms.

## Results and discussions

**Figure 2:**
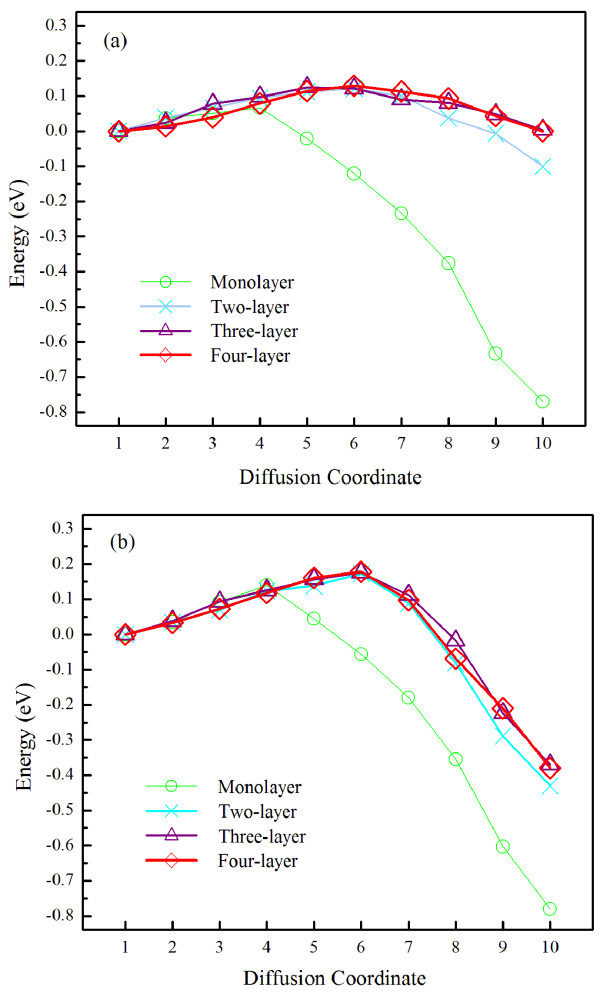
**Energy as a function of diffusion coordinate**. For (a) B-type steps and (b) A-type steps; the energy of the initial configuration of diffusion is taken to be zero.

In presenting the results, we first look at the overall energy barriers for the Al metal surface steps. Figure 2a shows the energy variation along the diffusion path, or diffusion coordinate, for B-type <110> steps. As discussed before, the exchange mechanism leads to a smaller diffusion barrier [25] and is the only diffusion mechanism investigated here. When the number of steps becomes two or three, the diffusion barrier converges to the 3D ES barrier. For B-type steps, the 3D ES barrier is 0.13 eV, and the 2D ES barrier (corresponding to a monolayer step) is 0.07 eV; for comparison, the diffusion barrier of an adatom on flat Al{111} is 0.05 eV, consistent with earlier reports [26]. It is interesting to note that this 3D ES barrier of 0.13 eV for Al is in sharp contrast to the 0.40 eV for Cu [8]; we have repeated the Cu calculations using ABINIT for consistency and obtained 0.41 eV, which is within the uncertainty of 0.01 eV. This result confirms our hypothesis that the 3D ES barrier of Al is small. Consequently, multiple-layer surface steps are unstable [13], and the corresponding characteristic length scale is much larger than 200 nm for the growth of Al nanorods [14]. For A-type <110> steps, Figure 2b shows that the 3D ES barrier is 0.18 eV and the 2D ES barrier is 0.14 eV. While the 3D ES barrier of A-type steps is larger than that of B-type steps, both are substantially smaller than the 0.40 eV of Cu B-type steps.

The results in Figure 2 are for diffusion from FCC to FCC sites. For B-type steps, when the diffusing adatom starts from a hexagonal-close-packed (HCP) site and ends at an FCC site, the 3D ES barrier is 0.14 eV. When the diffusing adatom starts from an FCC site and ends at an HCP site, the 3D ES barrier is 0.15 eV. When the diffusing adatom starts from an HCP site and ends at an HCP site, the 3D ES barrier is 0.15 eV. For an A-type step, when the diffusing adatom starts from an HCP site, the 3D ES barrier is reduced to 0.16 eV.

**Figure 3:**
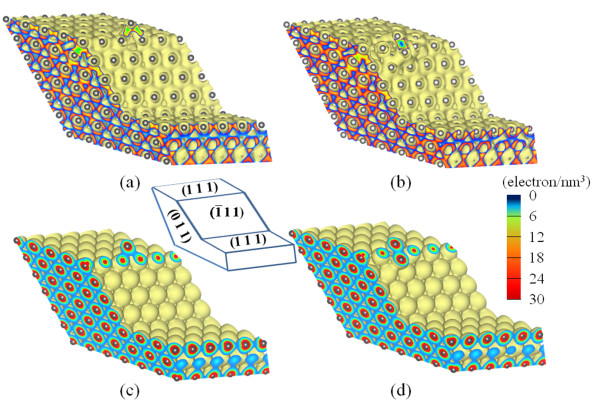
**Electron density distributions**. At the initial stage (a) and at the saddle point (b) of diffusion for Al and at the initial stage (c) and at the saddle point (d) of diffusion for Cu. The cross sections show the directional distribution with the covalent bonding between atoms in Al and the spherical charge distribution about a centered ion in Cu.

To gain insight of why the 3D ES barrier of Al is so much smaller than that of Cu, we compare the density of electrons in real space for the B-type step of four layers. As Figure 3a shows, there is substantial overlap of electrons between neighboring Al atoms and between Al adatom and its neighbors. This is also true when the Al adatom is at the saddle point (Figure 3b). In contrast, the overlap of electrons is substantially smaller in Cu (Figure 3c, d).

**Figure 4:**
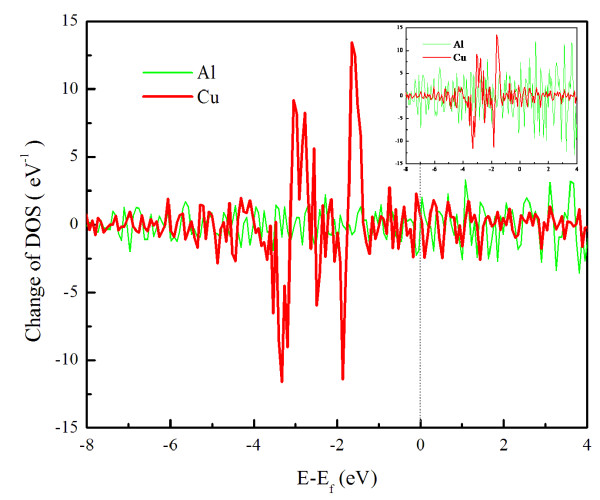
**Change of total DOS from initial configuration to saddle-point configuration**. It corresponds to steps in Figure 3; with Fermi level indicated by a dotted line.

Accompanying the electron density distribution in real space is its distribution in energy space. Figure 4 shows the change of total density of state (DOS) from the starting configuration to the saddle-point configuration of the diffusion process over the same four-layer B-type step. In comparison, fewer electrons move into higher energy states for Al than in Cu. Even when the DOS of Al is scaled by 11/3, this comparison is still valid as shown by the inset of Figure 4; Al has three valence electrons and Cu has 11. In covalent bonding, electrons move with bonds during diffusion and thereby can stay in low energy states. The covalent bonding of Al correlates with the smaller change of DOS.

Having linked a smaller 3D ES barrier of Al with intrinsic covalent bonding, we now discuss the impacts of this link. It is possible to *extrinsically *modify local degree of covalent bonding. Surfactants have been used to modify diffusion, as well as nucleation, on surfaces [27,28]. If a surfactant atom can modify the degree of local covalent bonding, it will change the 3D ES barrier. For example, the introduction of Si atoms to Cu steps may promote the local *sp *electronic bonding and thereby the local covalent bonding, but to qualify as a surfactant, the atom (such as Si) also has to float on surfaces. That is, in addition to effects of atomic size and bonding strength, the control of degree of covalent bonding derived from surfactants is a new effect. This new effect may lead to another mechanism of controlling surface morphologies during growth, say by physical vapor deposition. There is a large body of literature reporting Cu nanorods by PVD [2,3,19], but none on Al nanorods by PVD without substrate cooling (at least none that we are aware of), consistent with the difference of intrinsic 3D ES barriers of the two metals. If a surfactant extrinsically promotes local covalent bonding, growing Cu nanorods may turn into thin films. Similarly, if a surfactant extrinsically reduces the degree of local covalent bonding, growing Al thin films may turn into nanorods.

## Conclusion

In conclusion, we hypothesize that the lack of Al nanorods by PVD is due to the small 3D ES barrier of Al. Using *ab initio *calculations, we have shown that the 3D ES barrier of Al is indeed much smaller than that of Cu (0.13 versus 0.40 eV for the smaller barrier in each metal), confirming the hypothesis. Further, we have shown that the smaller 3D ES barrier of Al is the result of its covalent bonding. Finally, based on these two results, we suggest a new mechanism of surfactant application--that is, the change of degree of local covalent bonding through the introduction of surfactant atoms.

## Competing interests

The authors declare that they have no competing interests.

## Authors' contributions

SL carried out the calculations and analyses, and HH designed the project and participated in the analyses. All authors read and approved the final manuscript.
